# Comparison of the ventilation characteristics in two adult oscillators: a lung model study

**DOI:** 10.1186/s40635-019-0229-2

**Published:** 2019-03-12

**Authors:** Tetsuya Yumoto, Takahisa Fujita, Sunao Asaba, Shunsuke Kanazawa, Atsunori Nishimatsu, Hideo Yamanouchi, Satoshi Nakagawa, Osamu Nagano

**Affiliations:** 1 Advanced Emergency and Critical Care Medical Center, Okayama University Hospital, 2-5-1, Shikata-cho, Kita-ku, Okayama, 700-8558 Japan; 2 Center for Innovative and Translational Medicine, Kochi University Medical School, 185-1, Kohasu, Oko-cho, Nankoku, Kochi 783-8505 Japan; 3 Department of Disaster and Emergency Medicine, Kochi University Medical School, 185-1, Kohasu, Oko-cho, Nankoku, Kochi 783-8505 Japan; 4 Department of Critical Care and Anesthesia, National Center for Child Health and Development, 2-10-1, Okura, Setagaya-ku, Tokyo, 157-8535 Japan

**Keywords:** High-frequency oscillatory ventilation (HFOV), Adult oscillator, Inspiratory time % (IT%), Ventilation efficiency, Actual stroke volume

## Abstract

**Background:**

Two recent large randomized controlled trials did not show the superiority of high-frequency oscillatory ventilation (HFOV) in adults with ARDS. These two trials had differing results, and possible causes could be the different oscillators used and their different settings, including inspiratory time % (IT%). The aims of this study were to obtain basic data about the ventilation characteristics in two adult oscillators and to elucidate the effect of the oscillator and IT% on ventilation efficiency.

**Methods:**

The Metran R100 or SensorMedics 3100B was connected to an original lung model internally equipped with a simulated bronchial tree. The actual stroke volume (aSV) was measured with a flow sensor placed at the Y-piece. Carbon dioxide (CO_2_) was continuously insufflated into the lung model ($\dot{\mathrm{V}}$CO_2_), and the partial pressure of CO_2_ (PCO_2_) in the lung model was monitored. Alveolar ventilation ($\dot{\mathrm{V}}$A; L/min) was estimated as $\dot{\mathrm{V}}$CO_2_ divided by the stabilized value of PCO_2_. $\dot{\mathrm{V}}$A was evaluated with several stroke volume settings in the R100 (IT = 50%) or several airway pressure amplitude settings in the 3100B (IT = 33%, 50%) at a frequency of 6 and 8 Hz, a mean airway pressure of 25 cmH_2_O, and a bias flow of 30 L/min. Assuming that $\dot{\mathrm{V}}$A = frequency^*a*^ × aSV^*b*^, values of *a* and *b* were determined. Ventilation efficiency was calculated as $\dot{\mathrm{V}}$A divided by actual minute ventilation.

**Results:**

The relationship between aSV and $\dot{\mathrm{V}}$A or ventilation efficiency were different depending on the oscillator and IT%. The values of *a* and *b* were 0 < a < 1 and 1 < b < 2 and were different for three conditions at both frequencies. $\dot{\mathrm{V}}$A and ventilation efficiency were highest with R100 (IT = 50%) and lowest with 3100B (IT = 33%) for high aSV ranges at both frequencies.

**Conclusions:**

In this lung model study, ventilation characteristics were different depending on the oscillator and IT%. Ventilation efficiency was highest with R100 (IT = 50%) and lowest with 3100B (IT = 33%) for high aSV ranges.

**Electronic supplementary material:**

The online version of this article (10.1186/s40635-019-0229-2) contains supplementary material, which is available to authorized users.

## Background

Two recent large randomized controlled trials did not show the superiority of high-frequency oscillatory ventilation (HFOV) in adults with ARDS [1, 2]. These two trials had differing results, and possible causes could be the different oscillators used and their different settings, including mean airway pressure (MAP), frequency, and inspiratory time % (IT%). The difference in the MAP has been discussed and examined [3–6], and animal studies have shown that higher frequencies could improve the lung protective effect [7, 8]. However, other issues have not been focused on.

Because adult oscillators have no function to monitor actual SV (aSV), the aSV in the two trials (OSCILLATE and OSCAR) were not clear [1, 2]. However, some studies have reported that the aSV during HFOV could be higher than the anatomical dead space volume at low frequencies [8–10]. Recently, we have examined the effect of bias flow (BF) rate on ventilation efficiency using a lung model and reported that the ventilation efficiency improved with a BF of 30 L/min or more compared to that with a BF of 20 L/min or less in the R100 oscillator (Metran Co. Ltd., Kawaguchi, Saitama, Japan) at 8 Hz [11]. If the BF rate is inadequate, the required aSV consequently increases in order to obtain the same alveolar ventilation ($\dot{\mathrm{V}}$A) for a given frequency. Therefore, ventilation efficiency is one factor to determine the amount of required aSV.

## Methods

### Experimental setting

**Fig. 1:**
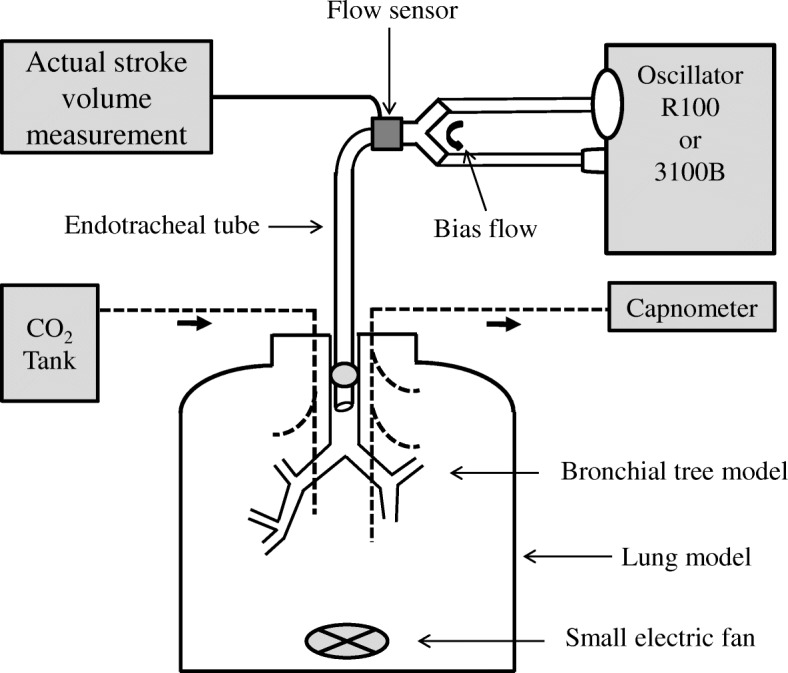
Schema of the experimental setting. For more information, see the text

The R100 or the SensorMedics 3100B (CareFusion, Yorba Linda, CA, USA) oscillator was connected to the lung model via an angle-type connector and an endotracheal tube (ETT) with an internal diameter of 8.0 mm and a length of 30 cm (Fig. 1). A microelectromechanical systems mass flow sensor (Siargo FS6022B150, Siargo Ltd., Santa Clara, CA, USA) was placed between the angle-type connector and the Y-piece for measuring aSV. The total dead space volume (VD) was approximately 110 mL, and the measured airway resistance was approximately 2, 5, and 8 cmH_2_O/L/sec with a flow of 10, 60, and 120 L/min, respectively. The common oscillator settings during the experiments were as follows: MAP of 25 cmH_2_O, BF of 30 L/min, and fraction of inspired oxygen of 0.21. The heated humidifier was turned off (hollow fiber type, R100) or removed from the circuit (chamber type, 3100B).

### Measurement of actual stroke volume

aSV was measured with a prototype stroke volume (SV) measurement system (Metran Co. Ltd., Kawaguchi, Saitama, Japan). In this system, the analog flow signal was sampled at 200 Hz and digitally integrated to determine expiratory SV every 1 s using a computer data acquisition system (LabView Ver.14, National Instruments, Austin, TX, USA). The mean value of 60 data measurements for 1 min was calculated as the aSV.

### Lung model

The lung model consisted of a 20-L airtight rigid plastic container internally equipped with a simulated bronchial tree model (KYOTO KAGAKU Co. Ltd., Kyoto, Japan) which had 3 to 7 steps of bifurcations to 20 segmental bronchial branches [11] (Fig. 1). The top of the ETT was located 3.5 cm from the carina. The total 20 L volume of the container accounts for an adiabatic static compliance of 19.3 mL/cmH_2_O (approximately equal to the severe ARDS [12]) due to gas compression. The container had two ports for gas insufflation and for gas sampling. A small electrical fan was placed horizontally on the bottom to assist gas mixing and to assure homogeneous CO_2_ distribution in the container.

### Measurement of alveolar ventilation

Carbon dioxide (CO_2_) was insufflated into the lung model at approximately 200 mL/min (float type area flowmeter), and continuous gas sampling was performed from the lung model using a capnometer (Life Scope TR, NIHON KOHDEN Co., Tokyo, Japan) (Fig. 1). The gas sampling rate of the capnometer was set at 200 mL/min. The partial pressure of CO_2_ (PCO_2_; mmHg) was monitored by the capnometer, and the stabilized value was recorded. The actual minute volume of insufflated CO_2_ ($\dot{\mathrm{V}}$CO_2_; mL/min) was calculated by the measurement of PCO_2_ using the capnometer when it was mixed with oxygen at 5 L/min (fixed-type flowmeter). This was performed before starting the experiment and was confirmed every 1 to 2 h. The $\dot{\mathrm{V}}$A was estimated by applying the alveolar ventilation equation (PCO_2_ = 0.863 × $\dot{\mathrm{V}}$CO_2_/$\dot{\mathrm{V}}$A, PCO_2_, and $\dot{\mathrm{V}}$A:BTPS, $\dot{\mathrm{V}}$CO_2_:STPD), though the equation was rearranged and used as $\dot{\mathrm{V}}$A = $\dot{\mathrm{V}}$CO_2_/PCO_2_ because all experiments were done under room temperature and dry conditions.

Although the SV can be set at up to 285 mL at a frequency of 6 Hz and 205 mL at a frequency of 8 Hz in the R100 oscillator, the measurable range of the flow sensor was limited. Therefore, $\dot{\mathrm{V}}$A was evaluated with the SV setting (sSV) from 80 to 180 mL (10 mL increments). The IT% was fixed at 50% in the R100 oscillator. In the 3100B oscillator, the IT% was set at 50% or 33%, and $\dot{\mathrm{V}}$A was evaluated with several setting amplitudes (Amp; 5 cmH_2_O increments, up to the maximum) at a frequency of 6 and 8 Hz. The minimum Amp setting was 20 cmH_2_O (IT = 50%, 6 Hz), 30 cmH_2_O (IT = 33%, 6 Hz and IT = 50%, 8 Hz), and 35 cmH_2_O (IT = 33%, 8 Hz). Assuming that $\dot{\mathrm{V}}$A = frequency^*a*^ × aSV^*b*^ [13], values of *a* and *b* were determined. The ratio of $\dot{\mathrm{V}}$A to the actual minute ventilation (a$\dot{\mathrm{V}}$E; aSV/1000 × frequency × 60, L/min) was calculated ($\dot{\mathrm{V}}$A/a$\dot{\mathrm{V}}$E) as an index for ventilation efficiency.

In both oscillators, $\dot{\mathrm{V}}$A was also evaluated with the targeted aSV of 80, 120, and 160 mL at 6 Hz and 80, 100, and 120 mL at 8 Hz, and $\dot{\mathrm{V}}$A/a$\dot{\mathrm{V}}$E was calculated (additional experiment). This additional experiment was for statistical analysis.

Each experiment was conducted five times.

### Statistical analysis

The statistical analysis was performed by BellCurve for Excel ver. 2.02 (SSRI Co. Ltd., Tokyo, Japan) using one-way analysis of variance followed by Tukey’s test. *P* < 0.05 was considered statistically significant. Curve fitting was also performed using the same software.

## Results

**Fig. 2:**
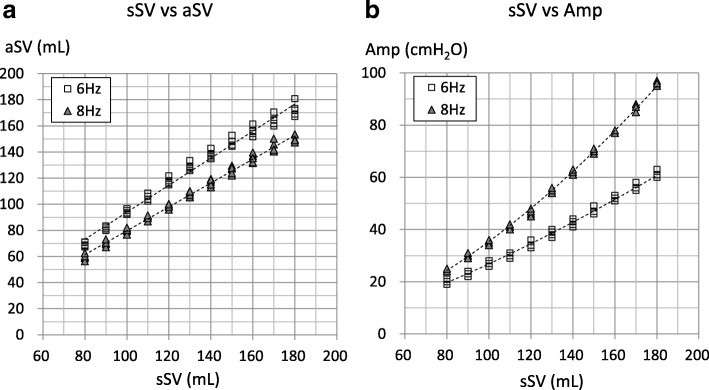
Relationship between setting stroke volume (sSV) and actual stroke volume (aSV) or airway pressure amplitude (Amp) in the R100. Markers indicate individual data. *Y*-axis in **b** is the airway pressure amplitude (Amp) displayed on the panel of the oscillator. Dotted lines in the **a** are first-order approximations. The coefficient of correlations (*R*) and *P* values are as follows: 6 Hz: *R* = 0.993, *P* < 0.001 and 8 Hz: *R* = 0.996, *P* < 0.001. Dotted curves in Fig. 2b are exponential approximations. The exponentials (coefficient of correlations (*R*), *P* values) are as follows: 6 Hz, 1.398 (*R* = 0.996, *P* < 0.001) and 8 Hz, 1.675 (*R* = 0.995, *P* < 0.001)

**Fig. 3:**
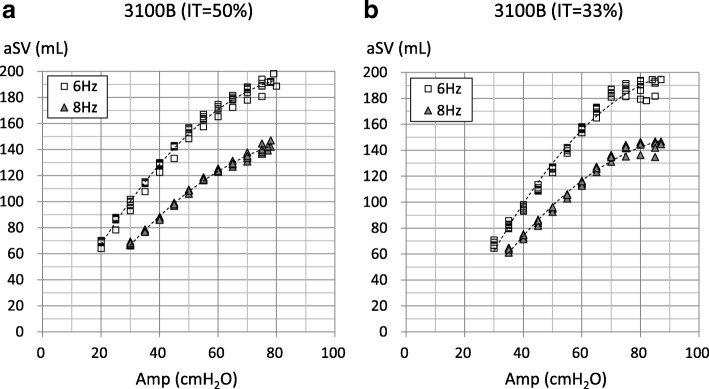
Relationship between airway pressure amplitude (Amp) and actual stroke volume (aSV) in the 3100B oscillator. Markers indicate individual data. *X*-axis is the airway pressure amplitude (Amp) displayed on the panel of the oscillator. **a** The results of IT = 50%. **b** The results of IT = 33%. The maximum Amp with the IT of 50% was 78.4 ± 1.4 cmH_2_O (mean ± SD) at 6 Hz and 77.4 ± 0.5 cmH_2_O at 8 Hz (**a**), and those with the IT of 33% were 84.2 ± 1.8 cmH_2_O at 6 Hz and 85.8 ± 1.1 cmH_2_O at 8 Hz (**b**). Dotted curves are second-order approximations. In **a** (IT = 50%), the coefficient of correlations (*R*) and *P* values are as follows: 6 Hz: *R* = 0.980 (*P* < 0.001) and 8 Hz: *R* = 0.987 (*P* < 0.001). In **b** (IT = 33%), *R* and *P* values are as follows: 6 Hz: *R* = 0.975 (*P* < 0.001) and 8 Hz: *R* = 0.977 (*P* < 0.001). In **a** (IT = 50%), there were no significant differences between the aSVs with an Amp of 70 and 75 cmH_2_O, 75 cmH_2_O and maximum Amp at 6 Hz, and between the aSVs with an Amp of 75 cmH_2_O and maximum Amp at 8 Hz. In the **b** (IT = 33%), there were no significant differences between all aSVs with an Amp ≥ 70 cmH_2_O at 6 Hz, and between all aSVs with an Amp ≥ 75 cmH_2_O and between the aSVs with an Amp of 70 and 75 cmH_2_O at 8 Hz

Figure 2 shows the relationships between aSV and sSV (Fig. 2a) and between Amp and sSV (Fig. 2b) in the R100 (IT = 50%). The aSV was linearly proportional to the sSV, and the Amp was correlated exponentially to the sSV at both frequencies. Figure 3 shows the relationships between Amp and aSV in the 3100B oscillator (Fig. 3a: IT = 50%, Fig. 3b: IT = 33%). The maximum Amp with the IT of 50% was 78.4 ± 1.4 cmH_2_O at 6 Hz and 77.4 ± 0.5 cmH_2_O at 8 Hz, and those with the IT of 33% were 84.2 ± 1.8 cmH_2_O at 6 Hz and 85.8 ± 1.1 cmH_2_O at 8 Hz (mean ± standard deviation). The aSV was well correlated to the Amp at both frequencies. aSV is generally plateaued with an Amp of 70 cmH_2_O or more at 6 Hz with the IT of 33% and with an Amp of 75 cmH_2_O or more with other settings.

**Fig. 4:**
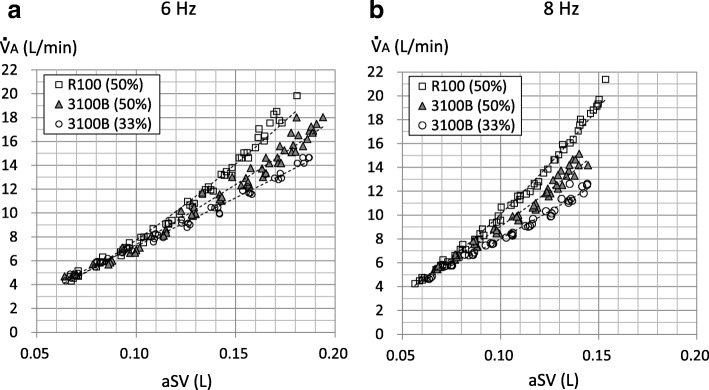
Relationship between actual stroke volume (aSV) and alveolar ventilation ($\dot{\mathrm{V}}$A). Markers indicate individual data of aSV (L) and $\dot{\mathrm{V}}$A (L/min). **a** The results at 6 Hz. **b** The results at 8 Hz. Dotted curves are exponential approximations. In **a** (6 Hz), the exponentials (coefficient of correlations (*R*), *P* values) are as follows: R100 (IT = 50%), 1.499 (*R* = 0.988, *P* < 0.001); 3100B (IT = 50%), 1.301 (*R* = 0.988, *P* < 0.001); and 3100B (IT = 33%), 1.125 (*R* = 0.991, *P* < 0.001). In the **b** (8 Hz), the exponentials (*R*, *P* values) are as follows: R100 (IT = 50%), 1.571 (*R* = 0.991, *P* < 0.001); 3100B (IT = 50%), 1.309 (*R* = 0.986, *P* < 0.001); and 3100B (IT = 33%), 1.140 (*R* = 0.991, *P* < 0.001)

Figure 4 shows the relationships between aSV (L) and $\dot{\mathrm{V}}$A (L/min) at 6 Hz (Fig. 4a) and 8 Hz (Fig. 4b). The indicated individual datasets in the 3100B are those with the Amp up to 70 cmH_2_O at 6 Hz with the IT of 33% and those with an Amp up to 75 cmH_2_O with other settings. $\dot{\mathrm{V}}$A was correlated exponentially to the aSV in three conditions at both frequencies. It appeared that $\dot{\mathrm{V}}$A was different in three conditions with the aSV generally greater than 140 mL (0.14 L) at 6 Hz and with the aSV generally greater than 100 mL (0.1 L) at 8 Hz (statistical analyses were not conducted). The values of *a* and *b* were 0 < a < 1 and 1 < b < 2 and were different in three conditions at both frequencies (Table 1). They were highest with R100 (IT = 50%) and lowest with 3100B (IT = 33%) at both frequencies.

**Table 1:** The results of the values for *a* and *b* according to the formula that $\dot{\mathrm{V}}$A(L/min) = frequency(cycle/min)^*a*^ × aSV(L)^*b*^

	Oscillator	VA = *k*1 × aSV^*b*^		*k*1 = frequency^*a*^	
	(IT%)	*k*1	*b*	Frequency (cycle/min)	*a*
6 Hz	R100 (50%)	242.50	1.4988	360	0.9329
	3100B (50%)	146.09	1.3009		0.8468
	3100B (33%)	95.43	1.1251		0.7744
8 Hz	R100 (50%)	374.26	1.5705	480	0.9597
	3100B (50%)	184.08	1.3089		0.8448
	3100B (33%)	110.41	1.1397		0.7620

The formula $\dot{\mathrm{V}}$A = *k* × aSV^*b*^ (*k* is constant) is an exponential approximation formula in Fig. 4. For more information, see the text

**Fig. 5:**
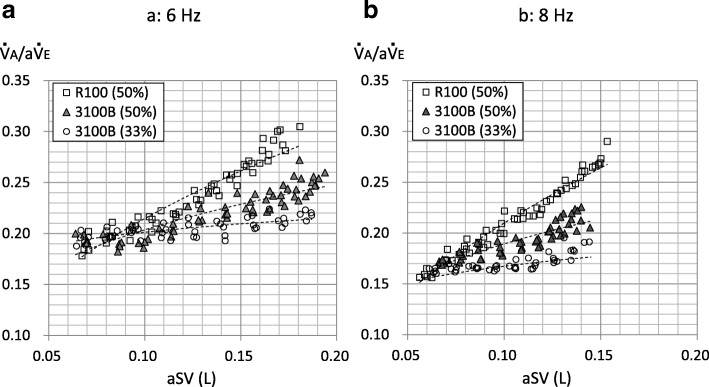
Relationship between actual stroke volume (aSV) and ventilation efficiency ($\dot{\mathrm{V}}$A/a$\dot{\mathrm{V}}$E). Markers indicate individual data of aSV and $\dot{\mathrm{V}}$A/a$\dot{\mathrm{V}}$E. **a** The results at 6 Hz. **b** The results at 8 Hz. Dotted lines are exponential approximations. In **a** (6 Hz), the exponentials (coefficient of correlations (*R*), *P* values) are as follows: R100 (IT = 50%), 0.4967 (*R* = 0.970, *P* < 0.001); 3100B (IT = 50%), 0.3005 (*R* = 0.914, *P* < 0.001; and 3100B (IT = 33%), 0.0948 (*R* = 0.715, *P* < 0.001). In the **b** (8 Hz), the exponentials (*R*, *P* values) are as follows: R100 (IT = 50%), 0.5705 (*R* = 0.986, *P* < 0.001); 3100B (IT = 50%), 0.3088 (*R* = 0.886, *P* < 0.001); and 3100B (IT = 33%), 0.1432 (*R* = 0.777, *P* < 0.001)

Figure 5 shows the relationships between aSV (L) and $\dot{\mathrm{V}}$A/a$\dot{\mathrm{V}}$E at 6 Hz (Fig. 5a) and 8 Hz (Fig. 5b). The indicated individual datasets in the 3100B oscillator are those with an Amp up to 70 cmH_2_O at 6 Hz with the IT of 33% and those with an Amp up to 75 cmH_2_O with other settings. $\dot{\mathrm{V}}$A/a$\dot{\mathrm{V}}$E was correlated exponentially to aSV in three conditions at both frequencies (Fig. 5). It also appeared that $\dot{\mathrm{V}}$A/a$\dot{\mathrm{V}}$E was highest in the R100 and lowest in the 3100B (IT = 33%) with the aSV generally greater than 140 mL (0.14 L) at 6 Hz and with the aSV generally greater than 100 mL (0.1 L) at 8 Hz (statistical analyses were not conducted).

*Additional experiment*: The measured aSVs with all targeted aSVs at both frequencies were not significantly different (Additional file 1). $\dot{\mathrm{V}}$A and $\dot{\mathrm{V}}$A/$\dot{\mathrm{V}}$E in the R100 (IT = 50%) were significantly higher than those in the 3100B (IT = 50%) with the targeted aSV of 160 mL at 6 Hz and with the targeted aSV of 100 and 120 mL at 8 Hz (Additional files 2 and 3). In the 3100B, $\dot{\mathrm{V}}$A and $\dot{\mathrm{V}}$A/$\dot{\mathrm{V}}$E were significantly higher with the IT of 50% than those with the IT of 33% for the targeted aSV of 120 and 160 mL at 6 Hz and for all targeted aSVs at 8 Hz (Additional files 2 and 3).

## Discussion

This study was performed with a constant MAP and a constant BF rate and showed that the oscillator and IT% influenced on the ventilation efficiency. We examined three conditions, i.e., R100 (IT = 50%), 3100B (IT = 50%), and 3100B (IT = 33%), with a frequency of 6 and 8 Hz in reference to the OSCILLATE and OSCAR trials [1, 2]. Several studies have measured aSV during adult HFOV [8–10, 14], though nobody has investigated ventilation efficiency using adult oscillators. Lower ventilation efficiency would increase the required aSV to obtain the same alveolar ventilation for a given frequency. Therefore, ventilation efficiency might be related to the benefit of HFOV.

The basic experimental setup in the current study was the same as the last report [11]. Therefore, we omitted the detailed discussion about the methods. Also, some limitations were discussed in that report [11]. One major limitation is that the absolute values of $\dot{\mathrm{V}}$A and $\dot{\mathrm{V}}$A/a$\dot{\mathrm{V}}$E cannot be applied to clinical situations mainly because of the incomplete bronchial tree model and rather small VD for an adult. Additionally, the lung model is far from a real lung, for example, it is rigid (not compliant) and it has only one big “alveolus.” We used the R100 (IT = 50%) at a single frequency (8 Hz) in the last report [11], though we used two oscillators, two IT%, and two frequencies in the current study. Our experimental setup is thought to be suitable in comparing the different events occurring outside the lung model (i.e*.,* in the oscillator circuit) when the events occurring in the lung model are thought to be identical with same aSV, same IT%, and same frequency. Therefore, it is thought that the effect of the oscillator with the same IT% and same frequency is applied to clinical situations. On the other hand, the events occurring in the lung model could differ by different frequency and different IT% even with the same aSV in the current study because of the different inspiratory and expiratory flow rate. It was reported that the gas mixing in the airway was mainly influenced by the central airway [15]. Therefore, our experimental setup likely reproduced the different events in the real lung caused by different frequency or by different IT% to some extent, though not completely. Accordingly, our experiment provides a relative indication of the effect of the oscillator and IT% on ventilation efficiency, but the absolute values we obtained should not be considered to be equal to what would be encountered in clinical practice.

In this study, the relationships between aSV and $\dot{\mathrm{V}}$A (Fig. 4) or $\dot{\mathrm{V}}$A/a$\dot{\mathrm{V}}$E (Fig. 5) are the most important. In clinical or experimental settings, the $\dot{\mathrm{V}}$A during HFOV had been determined as frequency^*a*^ × SV^*b*^ (the values for *a* and *b* were approximately 1 and 2, respectively) [13]. Therefore, we applied the power approximation curves to the relationships between aSV (the unit is L) and $\dot{\mathrm{V}}$A, and those were well fitted at both frequencies (Fig. 4), similar to the last report [11]. Furthermore, we calculated the exponential for the frequency (i.e., *a*) using cycle per minute as the unit of frequency, namely 360 cycle/min for 6 Hz and 480 cycle/min for 8 Hz (Table 1). The value for *a* was less than 1, and the value for *b* was between 1 and 2. Both values for *a* and *b* were highest in the R100 (IT = 50%) and lowest in the 3100B (IT = 33%) at both frequencies. This might reflect the different ventilation characteristics in the three conditions. According to the formula that $\dot{\mathrm{V}}$A = frequency^*a*^ × aSV^*b*^, the formula for ventilation efficiency would be as follows: $\dot{\mathrm{V}}$A/a$\dot{\mathrm{V}}$E = 1/frequency^(1−*a*)^ × aSV^(*b*−1)^. Therefore, we applied power approximation curves to the relationships between aSV (the unit is L) and $\dot{\mathrm{V}}$A/a$\dot{\mathrm{V}}$E. These were well fitted at both frequencies (Fig. 5), though we applied linear regression in the last report [11]. On the other hand, the formula of “frequency × SV^2^” (i.e., *a* = 1, *b* = 2) is well known as CO_2_ diffusion coefficient (DCO_2_) which is often used as an index of CO_2_ removal during neonatal HFOV [16]. However, our results showed that $\dot{\mathrm{V}}$A during adult HFOV would not follow DCO_2_.

In the next three paragraphs, we will discuss the possible mechanisms for the different ventilation characteristics in the three conditions in the following order: (1) effect of the IT% (50% vs 33% in 3100B), (2) effect of the oscillator with IT = 50% (R100 vs 3100B), and (3) effect of the frequency (6 Hz vs 8 Hz).

First, we will discuss the mechanisms of different ventilation characteristics due to different IT% for a given frequency in the 3100B oscillator. With the same aSV, changing IT from 50% to 33% increases the mean inspiratory flow rate by a factor of 1.5 and decreases the mean expiratory flow rate by a factor of 0.75. This could affect some specific ventilation mechanisms of HFOV in the lung. On the other hand, the gas regurgitation from the expiratory circuit to the inspiratory circuit during the expiratory phase of the oscillation could increase due to a longer expiratory time with lower IT%, and this might impair CO_2_ washout from the oscillator circuit. Yamada et al. investigated the effect of inspiratory and expiratory time (I:E) ratio on alveolar ventilation in dogs and reported no difference between the I:E ratio of 1:1 and 1:4 [17]. In their study, BF was completely aspirated through a thin catheter placed in the ETT. Gas regurgitation during the expiratory phase of the oscillation would not occur in such systems. Consequently, their study might indicate that the observed effect in our study would mainly arise from the differences of the oscillator circuit.

Second, we will discuss the mechanisms of different ventilation characteristics due to the different oscillator with the same frequency and same IT%. It is conceivable that the phenomena occurring in the lung model were mostly identical in this case. Therefore, the differences of $\dot{\mathrm{V}}$A and $\dot{\mathrm{V}}$A/a$\dot{\mathrm{V}}$E due to the oscillator with an IT of 50% would mainly arise from the different phenomena occurring in the oscillator circuit. Since the BF rate was constant in the current study, the differences in the oscillator circuit are thought to be the main cause. The circuit of the 3100B oscillator is narrow and short compared to that of the R100 oscillator. Furthermore, the R100 oscillator has a one-way valve at the end of the expiratory circuit to prevent gas regurgitation through the expiratory valve due to the negative airway pressure during the expiratory phase of the oscillation, though the 3100B does not have this valve. Such differences would change the detailed gas movement in the oscillator circuit, and CO_2_ washout from the oscillator circuit might change.

Finally, it was not the aim of this study to investigate the effect of the frequency on ventilation efficiency, though we think that several factors might be involved in this issue. With the same aSV and same IT%, changing frequency from 6 Hz to 8 Hz increases mean inspiratory and expiratory gas flow rates by a factor of 1.33. Therefore, some specific ventilation mechanisms of HFOV would be strengthened in the lung. On the other hand, the need for a higher BF rate would increase with higher frequency [11]. Therefore, the effect of the frequency on ventilation efficiency in our study would be influenced by the incomplete lung model and by the relatively insufficient BF rate with higher frequency. In the large animal study which showed the superiority of higher frequency for lung protection, the aSV became lower as frequency increased [8]. The fact that the frequency used in the OSCAR trial [2] was higher than that in the OSCILLATE trial [1] would be noteworthy, and its cause might be related to the different ventilation characteristics shown in the current study.

## Conclusions

In this lung model study, ventilation characteristics were different depending on the oscillator and IT%. Ventilation efficiency was highest with R100 (IT = 50%) and lowest with 3100B (IT = 33%) for high aSV ranges.

## Additional files

Additional file 1: Actual stroke volume (aSV) with targeted aSV. Bar graph indicates mean aSV (*n* = 5), and vertical bar indicates standard deviation. There are no significant differences between R100 (IT = 50%), 3100B (IT = 50%), and 3100 B (IT = 33%) with all targeted aSV at both frequencies. (PPTX 710 kb)

Additional file 2:Alveolar ventilation ($\dot{\mathrm{V}}$A) measured with targeted actual stroke volume (aSV). Bar graph indicates mean $\dot{\mathrm{V}}$A (*n* = 5), and vertical bar indicates standard deviation. The results of the statistical significance test in Additional file 2a (6 Hz) are as follows: R100 (IT = 50%) vs 3100B (IT = 50%): ns with aSV = 80 and 120, *P* < 0.001 with aSV = 160; R100 (IT = 50%) vs 3100B (IT = 33%): *P* < 0.001 with all aSV; 3100B (IT = 50%) vs 3100 B (IT = 33%): *P* < 0.05 with aSV = 80, *P* < 0.001 with aSV = 120, *P* < 0.01 with aSV = 160. The results of the statistical significance test in Additional file 2b (8 Hz) are as follows: R100 (IT = 50%) vs 3100B (IT = 50%): ns with aSV = 80, *P* < 0.001 with aSV = 100 and 120; R100 (IT = 50%) vs 3100B (IT = 33%): *P* < 0.001 with all aSV; 3100B (IT = 50%) vs 3100B (IT = 33%): *P* < 0.001 with all aSV. (PPTX 716 kb)

Additional file 3:Ventilation efficiency ($\dot{\mathrm{V}}$A/a$\dot{\mathrm{V}}$E) measured with targeted actual stroke volume (aSV). Bar graph indicates mean $\dot{\mathrm{V}}$A/a$\dot{\mathrm{V}}$E (*n* = 5), and vertical bar indicates standard deviation. The results of the statistical significance test in Additional file 3a (6 Hz) are as follows: R100 (IT = 50%) vs 3100B (IT = 50%): ns with aSV = 80 and 120, *P* < 0.001 with aSV = 160; R100 (IT = 50%) vs 3100B (IT = 33%): *P* < 0.001 with all aSV; 3100B (IT = 50%) vs 3100 B (IT = 33%): *P* < 0.01 with aSV = 80, *P* < 0.001 with aSV = 120, *P* < 0.05 with aSV = 160. The results of the statistical significance test in Additional file 3b (8 Hz) are as follows: R100 (IT = 50%) vs 3100B (IT = 50%): ns with aSV = 80, *P* < 0.001 with aSV = 100 and 120; R100 (IT = 50%) vs 3100B (IT = 33%): *P* < 0.001 with all aSV; 3100B (IT = 50%) vs 3100B (IT = 33%): *P* < 0.001 with all aSV. (PPTX 717 kb)

## Abbreviations

$$ \dot{\mathrm{V}} $$A: Alveolar ventilation
$\dot{\mathrm{V}}$A/a$\dot{\mathrm{V}}$E: Ratio of alveolar ventilation to actual minute ventilation
a$\dot{\mathrm{V}}$E: Actual minute ventilation
Amp: Airway pressure amplitude
ARDS: Acute respiratory distress syndrome
aSV: Actual stroke volume
BF: Bias flow
$\dot{\mathrm{V}}$CO_2_: Minute volume of insufflated CO_2_
CO_2_: Carbon dioxide
DCO_2_: CO_2_ diffusion coefficient
ETT: Endotracheal tube
HFOV: High-frequency oscillatory ventilation
I:E: Inspiratory and expiratory time ratio
IT: Inspiratory time
MAP: Mean airway pressure
PCO_2_: Partial pressure of CO_2_
sSV: Setting stroke volume
SV: Stroke volume
VD: Dead space volume
