# Supplementary figures and images for: Comparison of the ventilation characteristics in two adult oscillators: a lung model study
Source: Intensive Care Med Exp. 2019 Mar 12;7:15. doi: 10.1186/s40635-019-0229-2 (PMC6419651; doi:10.1186/s40635-019-0229-2)

## Slide 1
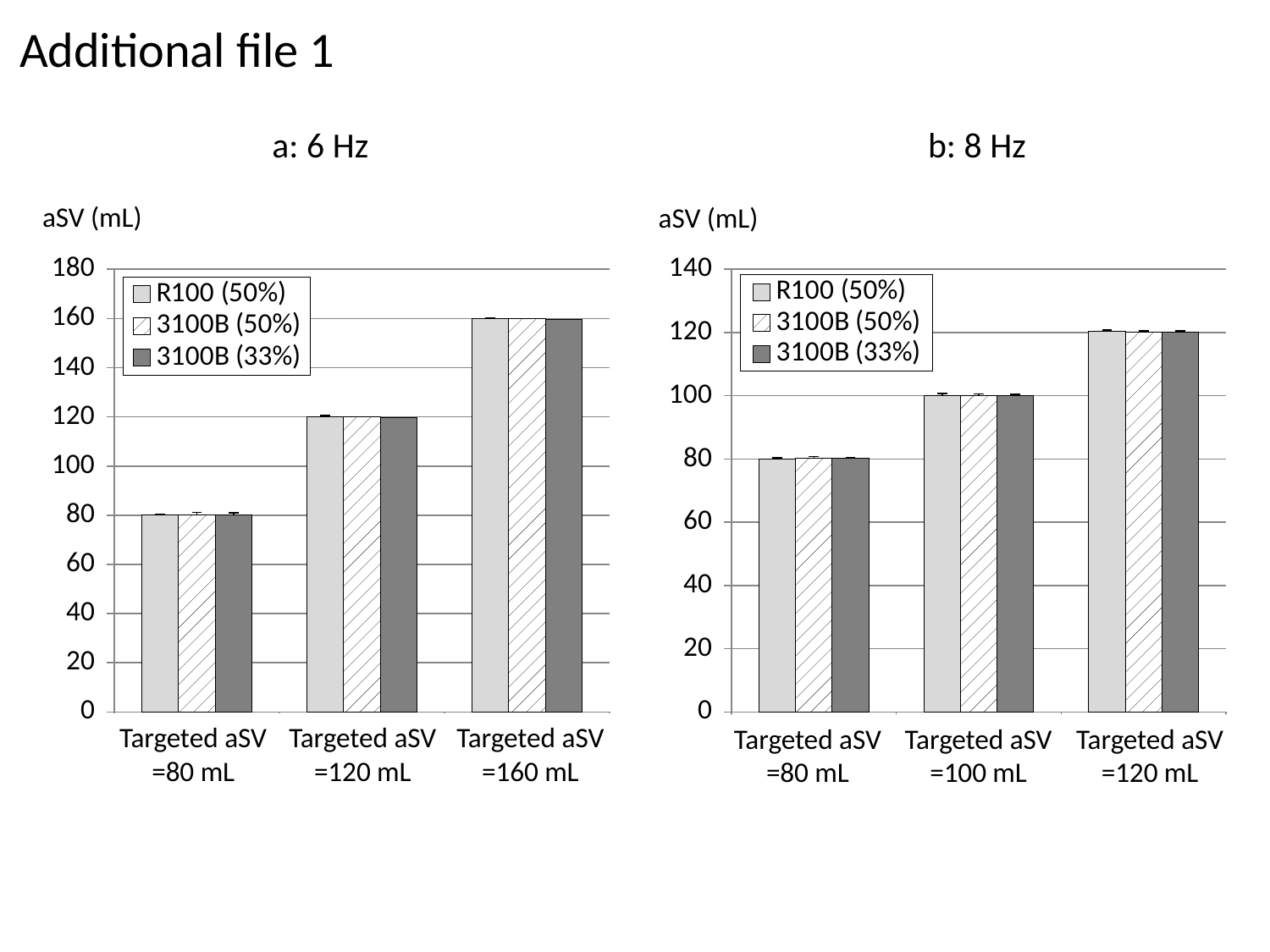

Supplement: Supplementary file 1 — Actual stroke volume (aSV) with targeted aSV. Bar graph indicates mean aSV (n = 5), and vertical bar indicates standard deviation. There are no significant differences between R100 (IT = 50%), 3100B (IT = 50%), and 3100 B (IT = 33%) with all targeted aSV at both frequencies. (PPTX 710 kb) [file 40635_2019_229_MOESM1_ESM.pptx]
