# Supplementary material for: Comparison of the ventilation characteristics in two adult oscillators: a lung model study
Source: Intensive Care Med Exp. 2019 Mar 12;7:15. doi: 10.1186/s40635-019-0229-2 (PMC6419651; doi:10.1186/s40635-019-0229-2)
Supplement: Supplementary file 2 — Alveolar ventilation ($\dot{\mathrm{V}}$V˙A) measured with targeted actual stroke volume (aSV). Bar graph indicates mean $\dot{\mathrm{V}}$V˙A (n = 5), and vertical bar indicates standard deviation. The results of the statistical significance test in Additional file 2a (6 Hz) are as follows: R100 (IT = 50%) vs 3100B (IT = 50%): ns with aSV = 80 and 120, P < 0.001 with aSV = 160; R100 (IT = 50%) vs 3100B (IT = 33%): P < 0.001 with all aSV; 3100B (IT = 50%) vs 3100 B (IT = 33%): P < 0.05 with aSV = 80, P < 0.001 with aSV = 120, P < 0.01 with aSV = 160. The results of the statistical significance test in Additional file 2b (8 Hz) are as follows: R100 (IT = 50%) vs 3100B (IT = 50%): ns with aSV = 80, P < 0.001 with aSV = 100 and 120; R100 (IT = 50%) vs 3100B (IT = 33%): P < 0.001 with all aSV; 3100B (IT = 50%) vs 3100B (IT = 33%): P < 0.001 with all aSV. (PPTX 716 kb) [file 40635_2019_229_MOESM2_ESM.pptx]

## Slide 1
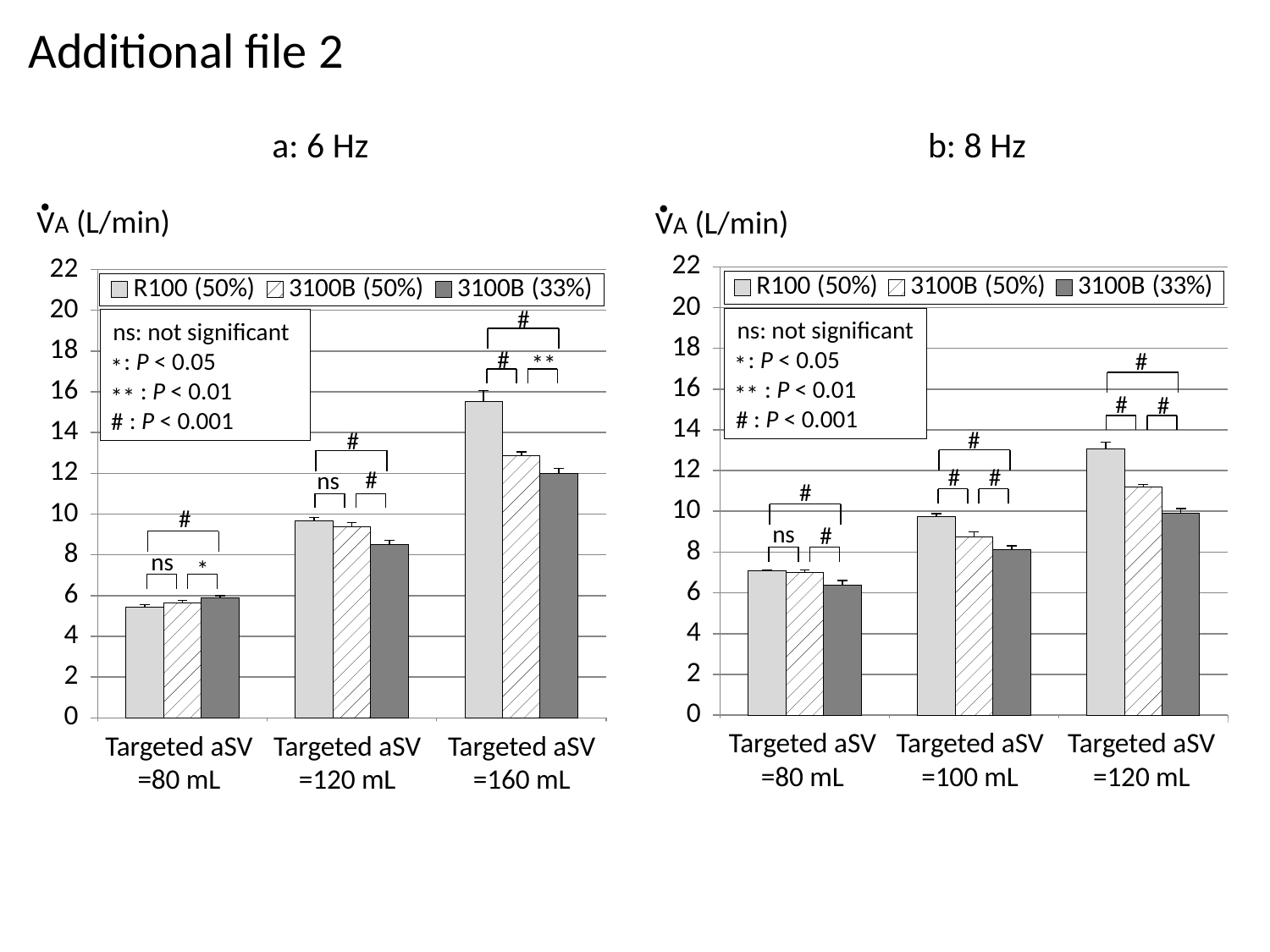

Additional file 2
a: 6 Hz
b: 8 Hz
・
VA (L/min)
・
VA (L/min)
### Chart
| Category | | | |
|---|---|---|---|
### Chart
| Category | | | |
|---|---|---|---|#
ns: not significant
 : P < 0.05
 : P < 0.01
 : P < 0.001
*
**
#
ns: not significant
 : P < 0.05
 : P < 0.01
 : P < 0.001
*
**
#
#
**
#
#
#
#
#
#
#
#
ns
#
#
ns
#
ns
*
Targeted aSV
=80 mL
Targeted aSV
=100 mL
Targeted aSV
=120 mL
Targeted aSV
=80 mL
Targeted aSV
=120 mL
Targeted aSV
=160 mL
